# Parvovirus-Induced Depletion of Cyclin B1 Prevents Mitotic Entry of Infected Cells

**DOI:** 10.1371/journal.ppat.1003891

**Published:** 2014-01-09

**Authors:** Richard O. Adeyemi, David J. Pintel

**Affiliations:** University of Missouri-Columbia, School of Medicine, Columbia, Missouri, United States of America; Wake Forest University, United States of America

## Abstract

Parvoviruses halt cell cycle progression following initiation of their replication during S-phase and continue to replicate their genomes for extended periods of time in arrested cells. The parvovirus minute virus of mice (MVM) induces a DNA damage response that is required for viral replication and induction of the S/G2 cell cycle block. However, p21 and Chk1, major effectors typically associated with S-phase and G2-phase cell cycle arrest in response to diverse DNA damage stimuli, are either down-regulated, or inactivated, respectively, during MVM infection. This suggested that parvoviruses can modulate cell cycle progression by another mechanism. In this work we show that the MVM-induced, p21- and Chk1-independent, cell cycle block proceeds via a two-step process unlike that seen in response to other DNA-damaging agents or virus infections. MVM infection induced Chk2 activation early in infection which led to a transient S-phase block associated with proteasome-mediated CDC25A degradation. This step was necessary for efficient viral replication; however, Chk2 activation and CDC25A loss were not sufficient to keep infected cells in the sustained G2-arrested state which characterizes this infection. Rather, although the phosphorylation of CDK1 that normally inhibits entry into mitosis was lost, the MVM induced DDR resulted first in a targeted mis-localization and then significant depletion of cyclin B1, thus directly inhibiting cyclin B1-CDK1 complex function and preventing mitotic entry. MVM infection thus uses a novel strategy to ensure a pseudo S-phase, pre-mitotic, nuclear environment for sustained viral replication.

## Author Summary

DNA viruses induce cellular DNA damage responses that can present a block to infection that must be overcome, or alternatively, can be utilized to viral advantage. Parvoviruses, the only known viruses of vertebrates that contain single-stranded linear DNA genomes, induce a robust DNA damage response (DDR) that features a cell cycle arrest that facilitates their replication. We show that the autonomous parvovirus MVM-induced cell cycle arrest is caused by a novel two-step mechanism that ensures a pseudo S phase, pre-mitotic, nuclear environment for sustained viral replication. A feature of this arrest is virally-induced depletion of the critical cell cycle regulator cyclin B1. Parvoviruses are important infectious agents that infect many vertebrate species including humans, and our study makes an important contribution to how these viruses achieve productive infection in host cells.

## Introduction

Parvoviruses are the only known viruses of vertebrates that contain single-stranded linear DNA genomes, and they present novel replicative DNA structures to cells during infection [1], [2]. Unlike the DNA tumor viruses, parvoviruses do not drive quiescent cells into S-phase [3]. However, following S-phase entry, cellular DNA polymerase, presumably DNA pol δ, converts the single stranded viral DNA genome into a double stranded molecule that serves as a template for transcription of the viral genes. The NS1 protein is the main viral replicator protein for the parvovirus minute virus of mice (MVM), interacting specifically with the viral genome to process its various replication intermediates. Parvoviruses establish replication factories in the nucleus (termed Autonomous Parvovirus-Associated Replication, or APAR, bodies) where active transcription of viral genes and viral replication takes place [4]–[6]. Viral replication induces a cellular DNA damage response which serves to prepare the nuclear environment for effective parvovirus takeover [7]–[11]. Following MVM infection, cellular genome replication soon ceases while viral replication continues for extended periods of time [12].

In order for viral replication to be sustained in infected cells, the cellular environment, including the replication machinery and raw materials for replication, must remain readily available. Thus, normal cell cycle progression must be altered. Parvoviruses employ varied mechanisms to disrupt normal cell cycle progression, sometimes in different ways depending on the type of cell infected [13]. Adeno-associated virus type 2 (AAV2) induces a S-phase block dependent upon Rep 78 nicking of cellular DNA and inhibitory stabilization of cell division cycle 25 A (CDC25A) [14]. B19 infection in semi-permissive cells causes a cell cycle arrest in G2 associated with accumulation of cyclins A, B1, and phosphorylated cyclin-dependent kinase 1 (CDK1) [15]. In the more permissive CD36 EPO cell line, B19 infection results in a G2 arrest primarily mediated by the viral NS1 protein through a mechanism that involves deregulation of the E2F proteins [16] independent of DNA damage signaling [11]. Minute virus of canines (MVC), a member of the *Bocavirus* genus of the *Parvoviridae* also induces a G2/M arrest that is associated with accumulation of cyclins and maintenance of inhibitory phosphorylation of CDK1 [17]. Interestingly, MVC G2 arrest is not dependent on the viral NS1 protein or on viral replication, but rather can be mediated by the viral genome *per se* - inoculation of UV-irradiated viral genomes was sufficient to induce a G2/M arrest. More recently, MVC was shown to induce a Structural Maintenance of Chromosome protein 1 (SMC1)-mediated S-phase arrest to enhance its replication [18]. MVM NS1 has been shown to inhibit cellular DNA replication, and effects on both cellular DNA integrity [19] and the DNA polymerase-α complex have been reported [20]. MVM infection has also been reported to cause a cell cycle arrest prior to mitosis [21]; however, the mechanism by which this occurs and its role in viral replication has not been fully characterized.

Two B-type cyclins exist in mammals, cyclin B1 and B2. Whereas cyclin B is an essential gene, cyclin B2-null mice develop normally, suggesting that B1 may compensate for cyclin B2's function in development [22]. Entry into mitosis requires both the accumulation of cyclin B1 and the activation of its associated CDK1 kinase *via* removal of its inhibitory phosphorylation [23]. This phosphorylation event is dependent on Wee1, which inhibits CDK1 by phosphorylating it on tyrosine 15; the CDC25 phosphatases antagonize Wee1 and activate CDK1 by removing the phosphorylation mark [24]–[28]. Thus, maintenance of CDK1 inhibitory phosphorylation is a major mechanism of G2-arrest in response to various DNA damaging agents [29], [30]. This is achieved mainly *via* the activated Chk1 kinase which inhibits the function of the CDC25 phosphatases, although Chk2 has been reported to inhibit these phosphatases as well [31], [32]. MVM induces a robust DDR in infected cells coordinated by ATM and characterized by phosphorylation of H2AX, Nbs1, Chk2 and p53 [7]. The DDR contributed to G2 arrest and was also required for robust viral replication in infected cells [7]. Surprisingly, the Chk1 kinase, which governs G2 arrest in response to myriad of DNA damage responses, was not activated to detectable levels during MVM infection [7]. Furthermore, the G2 cell cycle arrest observed in MVM-infected murine A9 cells was not a consequence of p53-mediated up-regulation of p21 [33].

In this report we further characterize the cell cycle perturbations that take place following infection with MVM. MVM infection presents the cell with sustained DNA damage signaling evidenced by increasing phosphorylation of H2AX throughout the course of infection [7]. But MVM infection also represents an atypical system in which two of the major players required for sustaining a G2 block in response to persistent DNA damage signaling, p21 and Chk1, are down-regulated or inactivated, respectively. How does viral infection sustain a cell cycle block in the absence of Chk1 activation or p21 up-regulation? We show here that the Chk2 protein was activated and recruited into MVM APAR bodies during infection. Chk2 activation was important at an early point in parvovirus infection, necessary to induce a transient S-phase block which was associated with CDC25A degradation. This early S-phase arrest was important for viral replication; however, Chk2 activation and CDC25A loss were not sufficient to sustain the marked G2 arrest seen following MVM infection. Rather, we have found that although the phosphorylation of CDK1 that normally inhibits entry into mitosis was lost as infection progressed, the MVM induced DDR resulted first in a targeted mis-localization and then a significant depletion of cyclin B1, thus directly inhibiting cyclin B1-CDK1 complex function and preventing mitotic entry. In this manner, MVM infection ensured a pseudo S-phase, pre-mitotic, nuclear environment for sustained viral replication.

## Results

### Chk2 activation mediated an S-phase arrest in MVM infected cells which facilitated viral replication

#### Chk2 was activated during MVM infection

The autonomous parvovirus MVM induces a DNA-damage response (DDR) in infected cells that results in cell cycle arrest prior to mitosis [7]. During this extended period the CDK inhibitor p21, which typically plays an important role in p53-mediated G2 arrest, was targeted for degradation in a proteasome-dependent manner [33]. This suggested that the MVM-induced cell cycle block was independent of the p53-p21 signaling axis.

**Figure 1:**
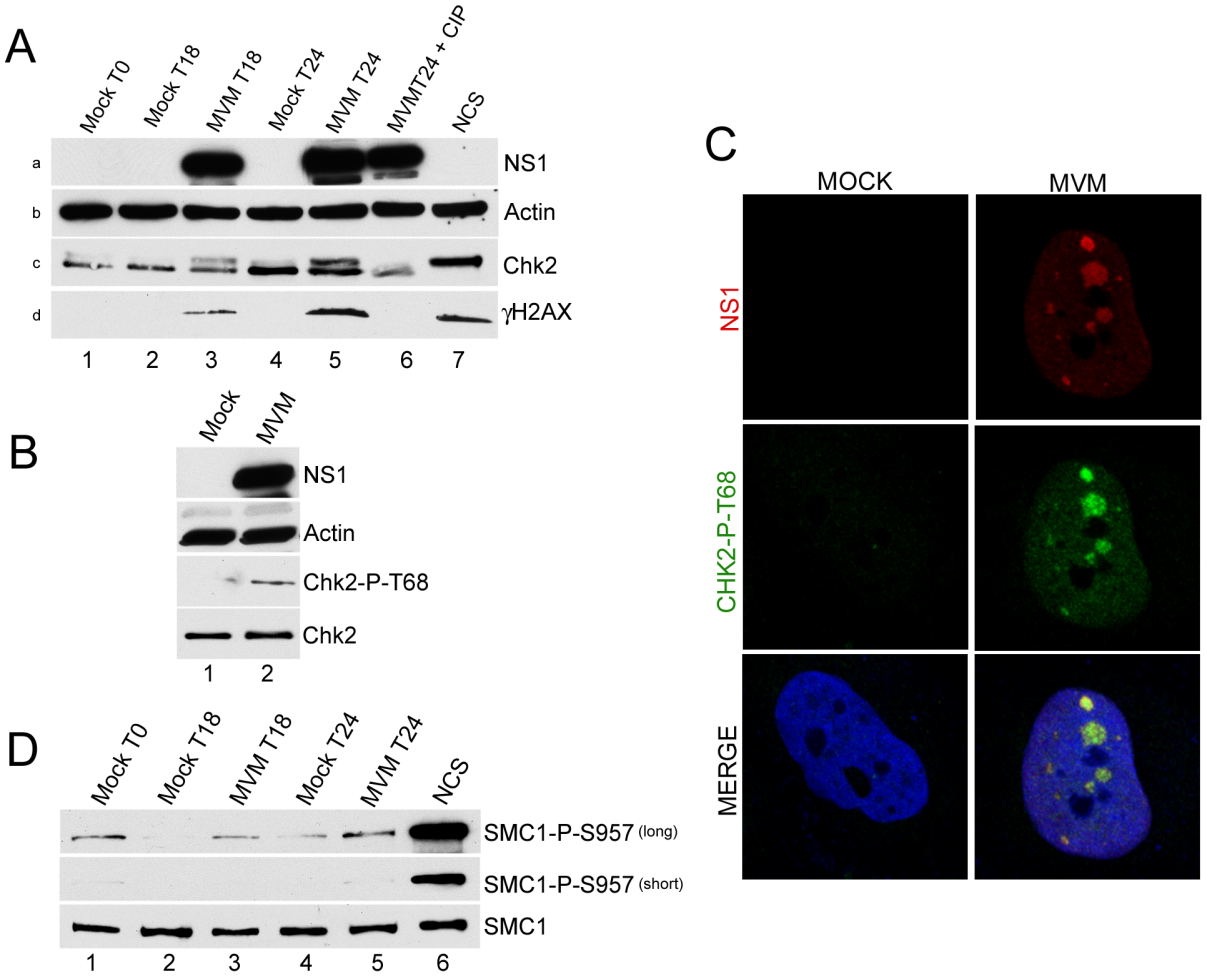
Chk2 but not SMC1 was activated during MVM infection. *(A) Time course of Chk2 activation following MVM infection of murine A9 cells.* A9 cells were para-synchronized in G0 as described in Materials and Methods. Cells were then mock-infected or infected with MVMp at an MOI of 10 at the time of release (T0, lane 1). As a positive control for Chk2 activation, A9 cells were treated with 150 ng/ml of the radiomimetic neocarzinostatin (NCS, lane 7) for 1 hour. Treatment with calf intestinal phosphatase (CIP, lane 6) was done for 1 hour at 37°C. Cells were harvested at the indicated time points after release, lysed in modified RIPA buffer. Protein content was measured using Bradford assay and equal amounts of protein were loaded in each well for immunoblotting. Western blot analysis was carried out using antibodies directed against NS1 (panel a), Chk2 (panel c) and H2AX phosphorylated on serine 139 (γH2AX, panel d). Equal loading was confirmed by blotting for actin (panel b). *(B) Chk2 activation following MVM infection in permissive human NB324K cells.* NB324K cells were mock infected or infected with MVM at an MOI of 10. Cells were harvested 24 hours post infection. Western blot analysis using antibodies directed against NS1, Actin, Chk2 phosphorylated on threonine 68 (Chk2-P-T68) and total Chk2 protein (Chk2) as indicated is shown. *(C) Activated Chk2 localized within MVM APAR bodies.* NB324K cells were infected for 24 hours before fixation and processing for immunofluorescence. APAR bodies were detected with antibodies to NS1. Nuclei were stained with TOPRO-3. Staining with an antibody to phosphorylated Chk2, observed only in infected cells, was prominent in distinct foci which co-localized with APAR bodies and also in a pan-nuclear pattern (merge panel). All images were captured using an objective of 63×. *(D) SMC1 is not significantly activated following MVM infection of murine A9 cells.* Lysates from Figure 1A were blotted with antibodies directed against total SMC1 protein (SMC1) and SMC1 protein phosphorylated on serine 957 (SMC1-P-S957). Short and long exposures of the same blot are shown.

As described above, Chk1 and Chk2 are additional critical downstream checkpoint proteins known to regulate cell cycle progression. In MVM infected, but not mock-infected, murine cells we found that Chk2 exhibited the altered electrophoretic mobility associated with activation (Figure 1A, panel c, compare lanes 3 & 5 to lanes 2 and 4). Chk2 activation increased as the infection progressed (Figure 1A, panel c, compare lanes 5 to 3), and could be reversed by treatment with calf intestinal phosphatase (Figure 1A, panel c, lane 6). Infection did not result in a total shift of the Chk2 species into the slower migrating form, as was found in cells treated with the radiomimetic neocarzinostatin (NCS) (Figure 1A, panel c, lane 7), likely because during infection of a para-synchronous population not all cells enter S-phase and support MVM replication in a perfectly uniform manner. MVM infection was confirmed by NS1 expression (Figure 1A, panel a, lanes 3, 5, 6), and an ongoing MVM-induced DDR was also confirmed by robust phosphorylation of H2AX on serine 139 (γH2AX) (Figure 1A, panel d, compare lanes 3 and 5 to lane 6) that was similar, or higher (at 24 h pi), than that observed following NCS treatment (Figure 1A, panel d, compare lane 5 to 7).

Chk2 activation is characterized by its phosphorylation on threonine 68 [34]. Because of species-specific antibody restrictions, this phosphorylation marker was examined in MVM-infected human NB324K cells. (MVM induces a DDR in the permissive NB324K cell line that is indistinguishable to that induced in murine cells [7]). 24 hours after infection, phosphorylation of Chk2 on threonine 68 was clearly apparent (Figure 1B, lane 2; phosphorylated human Chk2 did not exhibit altered electrophoretic mobility under these gel conditions). Phosphorylated Chk2 also localized to MVM APAR bodies, sites of ongoing virus replication. As shown in Figure 1C, MVM infected, but not mock infected, NB324K cells showed reactivity with the anti-Chk2 T68 antibody (Figure 1C, middle panel). Chk2 T68 staining was increased in the entire nucleus but showed greater staining intensity and localization within nuclear APAR bodies, identified by the presence of MVM NS1 (Figure 1C, top panel). The specificity of this antibody was validated by confirming reactivity with NCS-activated samples and the loss of this reactivity upon further addition of the Chk2 inhibitor (data not shown). We did not observe redistribution of the total Chk2 protein to APAR bodies in infected cells (data not shown), suggesting that only the activated Chk2 protein was re-localized.

We could not detect activation of Chk1 during MVM infection of NB324K cells in these experiments (data not shown), as also previously reported for MVM infection of murine A9 cells [7], arguing against a major role for this kinase in MVM-induced cell cycle arrest. Similarly, we failed to observe activation, as detected by its phosphorylation, of SMC1, a chromosomal protein that is a component of the cohesin complex which has been implicated in cell cycle arrest particularly in S-phase. SMC1 was recently shown to be important for S-phase arrest in bocavirus infected cells [18]. However, A9 cell extracts from the same experiment shown in Figure 1A exhibited only a slight increase in SMC1 phosphorylation above mock-infected cell levels at each time point tested (detectable only upon extended exposure, Figure 1D, top panel, compare lanes 2 & 3 or lanes 3 & 4), and these levels never exceeded the background seen as a consequence of the synchronization procedure (Figure 1D, top panel, lane 1). MVM infection did not alter amounts of total SMC1 protein (Figure 1D, bottom panel). As expected, control treatment with NCS resulted in significant phosphorylation of SMC1 (Figure 1B lane 6). These results suggested that MVM-induced murine cell cycle arrest likely occurs independently of high levels of activated SMC1.

#### Chk2 activation during MVM infection resulted in a transient S-phase arrest associated with degradation of CDC25A

**Figure 2:**
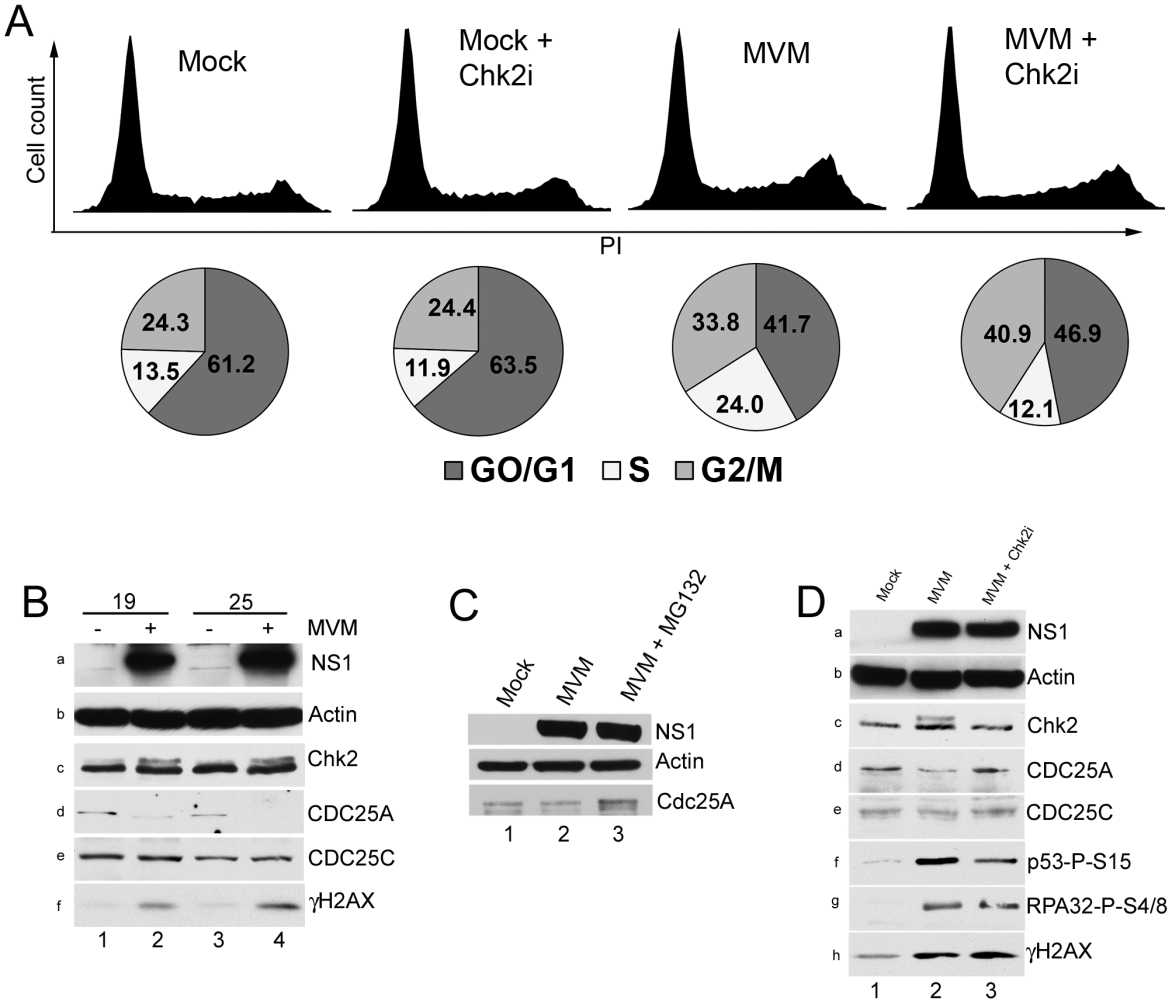
Chk2 activation leads to a transient S-phase accumulation mediated by loss of CDC25A. *(A) Reversal of S-phase accumulation following Chk2 inhibition.* FACS analysis was carried out as described in Materials and Methods. Cells were pre-treated for one hour with 10 µM Chk2 inhibitor II (Chk2i) or DMSO vehicle before being either mock-infected or infected with MVMp at a MOI of 10 for 24 hours. Representative histograms showing cell counts plotted against propidium iodide (PI) intensity are shown. Histograms are not drawn to scale. The percentage of cells in each cycle stage was quantified using Summit software and shown as pie charts. Percentages are averages of three independent experiments. *(B) CDC25A degradation during infection.* Paraynchronized A9 cells were mock-treated or infected with MVM at an MOI of 10. Samples were taken at either 19 or 25 h pi as shown, and immunoblotted using antibodies directed against NS1 (panel a), Actin (panel b), total Chk2 (panel c), CDC25A (panel d), CDC25C (panel e) and γH2AX (panel f). *(C) CDC25A is stabilized by proteasome inhibition.* Parasyncrhonous mock-treated cells (harvested at 24 h after release) and cells infected with MVM at an MOI of 10 in the presence and absence of MG132 (added at 18 h before harvest at 24 hpi) were immunoblotted with the indicated antibodies. *(D) Chk2 inhibition prevents CDC25A loss.* A9 cells were pretreated with 10 µM of Chk2 inhibitor II or DMSO control one hour before being either mock-infected (DMS0) or MVM infected at an MOI of 10. At 24 h pi, cells were harvested and processed as described in Figure 2B. Additionally, lysates were blotted with antibodies directed against p53 phosphorylated on serine 15 (p53-P-S15, panel f) and RPA32 phosphorylated on serine 4/8 (RPA32-P-S4/8, panel g).

**Figure 3:**
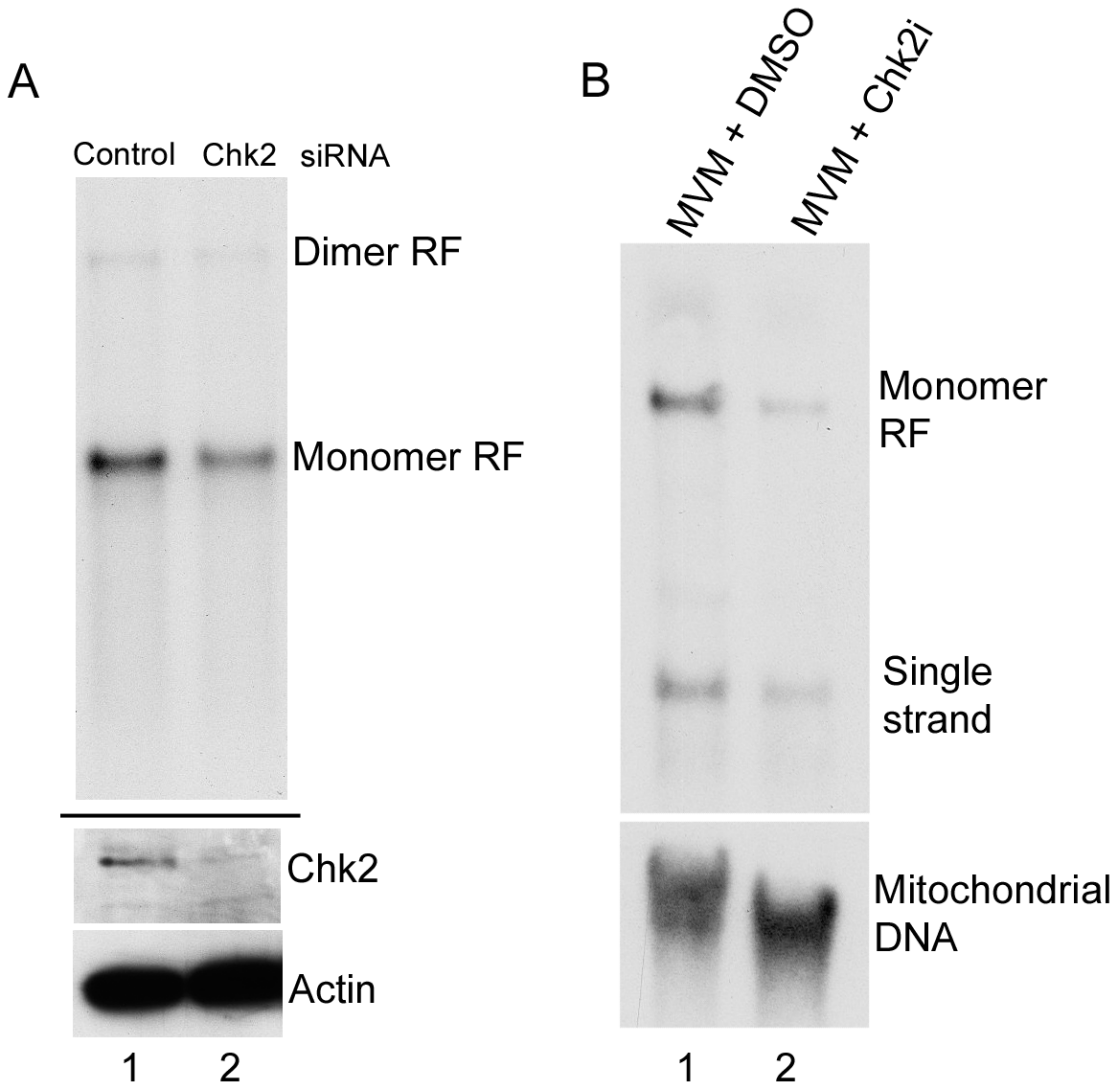
Chk2-mediated S-phase arrest facilitates MVM replication. *(A) siRNA depletion of Chk2 reduces MVM replication.* A9 cells were transfected twice with 40 nM of control siRNA (lane 1) or siRNA against Chk2 (lane 2) as described in the Materials and Methods. At the time of release from para-synchronization, cells were infected at an MOI of 2. 24 hours later cells were harvested and split in two for Southern and western blotting. Top panel shows a Southern blot demonstrating reduced monomer replicative form (RF, compare lane 2 to 1). Bottom panel shows a western blot showing Chk2 knockdown (compare lane 2 to 1). Actin was blotted as a loading control. There was a two-fold reduction in viral replication following Chk2 depletion by siRNA *(B) Chk2 inhibition reduces MVM replication.* A9 cells were pretreated with DMSO (lane 1) or Chk2 inhibitor II (Chk2i, lane 2)) before infection at an MOI of 2. 24 hours later cells were harvested and Southern blotting was performed. Bottom panel was probed using a mitochondrial DNA probe for standardization. There was a 4.5 fold reduction in viral replication in the presence of Chk2 inhibitor compared with DMSO treated cells.

MVM infection of asynchronous murine A9 cells resulted in a substantial increases in the amount of cells in both S and G2 phases compared to non-infected controls (from 13.5% to 24% for S phase and 24% to 42% for G2 phase respectively, Figure 2A, compare mock to MVM). In these experiments 70 to 80% of the cells were infected as determined by immunofluorescence for NS1 at this time point (data not shown). Addition of Chk2 inhibitor II affected infected cell cycle progression, but not by significantly reducing accumulation in G2. Rather, inhibitor treatment resulted in a substantial reduction in the amount of cells that accumulated in S-phase (from 24% to 12%, Figure 2A) without altering the cell cycle distribution of uninfected cells. This result suggested that in MVM infected cells activated Chk2 played a role during S-phase transition, rather than mediating cell cycle arrest in G2. The inhibitor was found to induce a modest increase in the percentage of cells resident in G2 (from 34% to 41%, Figure 3A), consistent with transition from an earlier transient block. More cells remained in G1 phase than expected for reasons that are not yet clear. An S-phase arrest during MVM infection has been noted previously by others [21].

Two parallel pathways have been primarily implicated in cell cycle arrest in S-phase following DNA damage – the Nbs1/SMC1 pathway and the ATM/Chk1-Chk2/CDC25A pathway [35]. Since inhibition of Chk2 kinase activity diminished the percentage of infected cells in S-phase, and because we failed to observe significant increases in SMC1 phosphorylation above background levels, we examined downstream events in the Chk2 signaling pathway. Both Chk1 and Chk2 activation have been implicated in CDC25A degradation following ionizing radiation [35]–[37]. As can be seen in Figure 2B, there was a significant loss of CDC25A in MVM infected A9 cells beginning at 19 h pi, when virus replication (as evidenced by NS1 expression) became prominent, and continued through 25 hours post infection (h pi) (Figure 2B, panels a and d, compare lanes 2 and 4 with lanes 1 and 3). H2AX phosphorylation confirmed an ongoing MVM-induced DDR (Figure 2B, panel f, lanes 4 and 6). The Chk2 mobility shift indicative of Chk2 activation was not clearly visible until 19 h pi in the experiment shown (Figure 2B, panel c, lanes 2 and 4), although we have observed it at earlier time points in other experiments (data not shown). We did not observe a reduction in the levels of the related protein CDC25C (Figure 2B, panel e), suggesting that the loss was specific to CDC25A. The loss of CDC25A could be substantially reversed by treatment with MG132 (Figure 2C, compare lanes 2 and 3), indicating that it had been targeted to the proteasome.

Treatment with Chk2 inhibitor II, which prevented the activation of Chk2 (as evidenced by the loss of Chk2 with an altered mobility; Figure 2D, panel c, compare lanes 2 and 3), prevented the reduction in CDC25A levels observed following MVM infection of either para-synchronous (Figure 2D, panel d, compare lanes 2 to 3), or asynchronous (data not shown) A9 cells, without affecting the levels of the related CDC25C protein (Figure 2D, panel e). The Chk2 inhibitor did not substantially affect accumulation of NS1 (Figure 2D, panel A), nor did it inhibit the MVM-induced DDR as indicated by levels of phosphorylated RPA32 and γH2AX (Figure 2D, panels g and h), suggesting that it performed its function subsequent to the initiation of genome replication. However, we did observe a reduction in activated p53 levels (Figure 2D, panel f, lanes 2 and 3), which may have been due to the previously reported role of Chk2 in phosphorylating p53 [38]. These results suggested that Chk2 activation resulted in reduced levels of CDC25A during MVM infection, as has been observed following treatment with ionizing radiation [36].

#### The Chk2-mediated S-phase arrest facilitated MVM replication

siRNA knockdown of Chk2 in A9 cells resulted in significant depletion of Chk2 protein in infected cells compared to control siRNA treated cells (Figure 3A, lower panel, compare lane 1 and 2). This resulted in an approximate two-fold reduction in MVM replication compared to cells treated with control siRNA (Figure 3A, upper panel, compare lane 1 and 2). Pre-treatment of asynchronously growing cells with the Chk2 inhibitor resulted in at least a four-fold reduction in accumulation of monomer replicative forms (Figure 3B, lane 2) compared to vehicle treated cells (Figure 3B, lane 1). In these experiments, NS1 accumulated levels were not drastically reduced even though there was reduced replication (data not shown, see also Figure 2B - lanes 2 and 3) which was likely due to the very stable nature of the NS1 protein [39]. Taken together, these results suggested that activation of Chk2 during infection induced a transient S-phase block which is important for MVM replication. It remains possible that the Chk2 may have other effects leading to enhancement of MVM replication.

### The MVM-induced G2/M cell cycle block featured initial mis-localization and subsequent loss of cyclin B1

#### MVM infection induced a sustained pre-mitotic block even though the inhibitory phosphorylation of CDK1 was lost

In addition to a transient block within S-phase, MVM infection results in an essentially complete block to the entry of infected cells into mitosis [21]. Whereas CDK2, which is mainly activated by CDC25A [40], plays important roles in S-phase progression, transit from G2 to mitosis in normal cycling cells is governed by activity of the CDK1 (also called cdc2) kinase in complex with its mitotic cyclin B1 [23]. This kinase can be subject to various regulatory mechanisms in response to DNA damaging drugs and during certain viral infections. In normal cycling cells in the absence of DNA damage, the Wee1 kinase phosphorylates CDK1 on Tyr 15 rendering it inactive. When cells cycle to the G2/M border, it is primarily the CDC25C phosphatase that removes this inhibitory phosphorylation, thus promoting activation of CDK1 and mitotic entry [40]. In order to determine the mechanism of G2-arrest following MVM infection, we first examined the activity and phosphorylation status of CDK1 over the course of infection.

**Figure 4:**
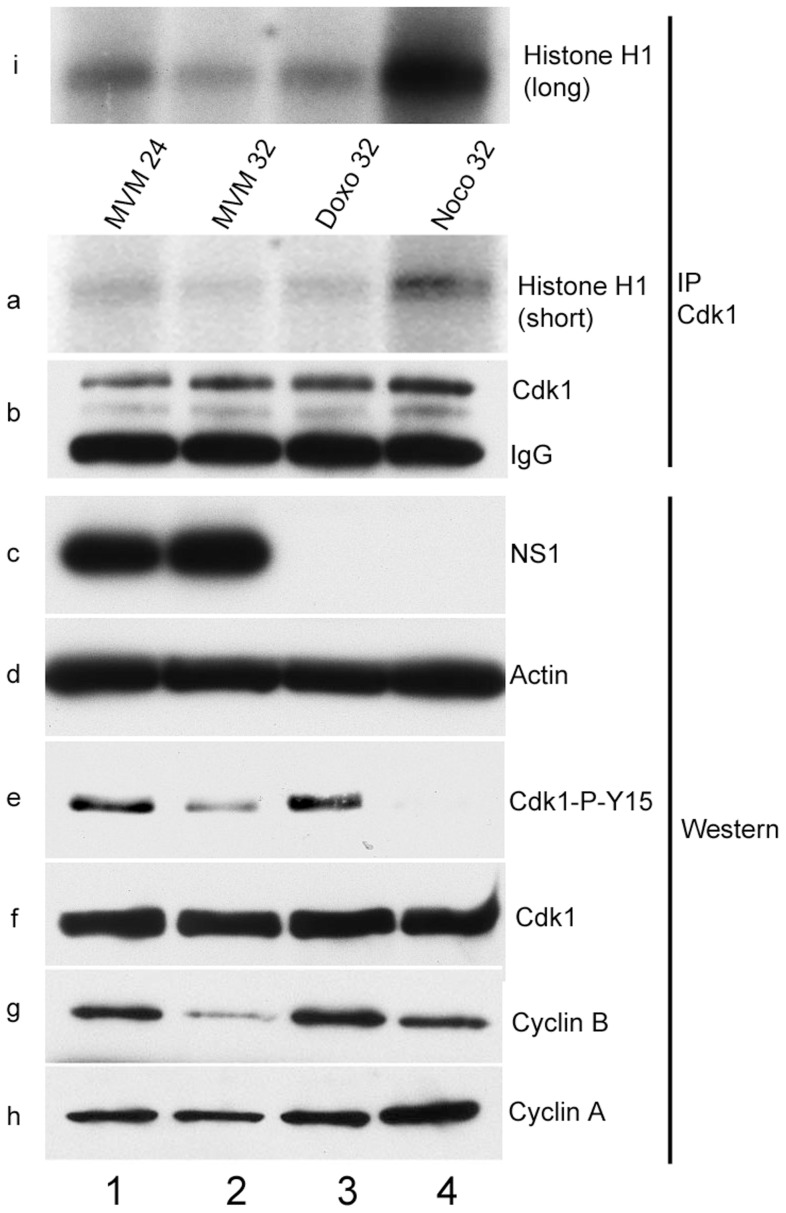
Inhibitory phosphorylation of CDK1 is transient during infection even though cells remain blocked in G2. Para-synchronized A9 cells were infected for the indicated time points. Control cells were treated with 200 nM doxorubicin (doxo, lane 3) or 150 ng/ml of nocodazole (noco, lane 4) 16 hours post release (just after S-phase entry) and harvested at the indicated time points. Cells were lysed in IP kinase buffer as described in materials and methods. Equal amount of lysates were immunoprecipitated with 4 µg of CDK1 antibody and used for kinase assays using histone H1 as substrate. Panel b shows amounts of immunoprecipitated CDK1. Autoradiograms of phosphorylated histone H1 are shown in panels a (short exposure) and i (long exposure). Input samples were blotted with antibodies directed against NS1 (panel c), actin (panel d), total CDK1 (panel f), CDK1 phosphorylated on tyrosine 15 (CDK1-P-Y15, panel e) and cyclins A (panel h) and B (panel g).

CDK1 kinase assays using histone H1 as a substrate demonstrated that at both early (24 h pi) and late (32 h pi) time points following MVM infection, CDK1 activity was reduced, to levels seen following control doxorubicin treatment which blocks cells in G2 (Figure 4, panels a and i, compare lanes 1 and 2 with lane 3). Cells treated with the microtubule inhibitor nocodazole, which traps cells in mitosis, and have high levels of CDK1 activity [41], exhibited high levels of histone H1 phosphorylation (Figure 4, panels a and i, lane 4). These results demonstrated that a significant portion of MVM infected cells were blocked prior to mitosis at these time points. As expected, the nocodazole treated samples resulted in undetectable levels of the inhibitory CDK1 phosphorylation on Y15 (since the cells have proceeded into mitosis) (Figure 4, panel e, lane 4); while doxorubicin treatment, which causes accumulation of cells in G2/M similar to that seen as a result of MVM infection, resulted in the persistence of the inhibitory phosphorylation of CDK1 (Figure 4, panel e, lane 3). The phosphorylation status of CDK1 in MVM infected cells was unexpected. At 24 h post MVM infection, CDK1 Y15 inhibitory phosphorylation was significant and comparable to what was observed following doxorubicin treatment (Figure 4, panel e, compare lanes 1 and 3). However, surprisingly, by 32 h pi, even though cells were arrested and CDK1 kinase activity was reduced even further (Figure 4, panels a and i, compare lanes 1 and 2), the inhibitory phosphorylation of CDK1 was significantly reduced (Figure 4, panel e, compare lanes 1 and 2) - a state normally associated with cell cycle progression. This suggested that the cell cycle block seen at later times during MVM infection may have been the result of another mechanism. Expression of NS1 confirmed ongoing viral infection (Figure 4, panel c, lanes 1 and 2).

#### Virus-mediated down-regulation of cyclin B1 prevented mitotic entry

**Figure 5:**
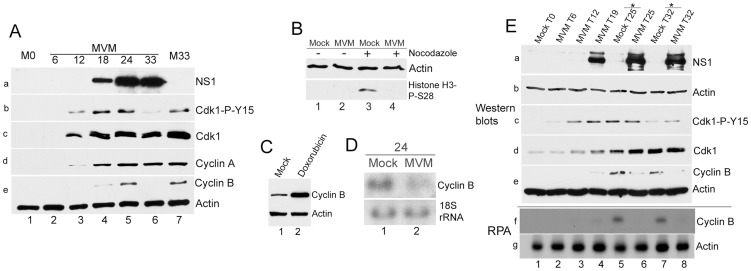
Virus-mediated downregulation of cyclin B1 RNA prevents mitotic entry. *(A) Cyclin B1 loss late in MVM infection.* Time course of MVM infection of para-synchronized A9 cells. Mock cells were harvested at the time of release (M0, lane 1) or 33 hours post release (M33, lane 7). Western blotting was performed as described in Figure 4, using antibodies directed against NS1 (panel a), CDK1-P-Y15 (panel b), total CDK1 (panel c), cyclin A (panel d), and cyclin B1 and actin (panel e). *(B) Infected cells do not proceed into mitosis.* Nocodazole trap assay was performed as described in Materials and Methods. Cells were blotted using antibodies against actin and histone H3 phosphorylated on serine 28 (Histone H3-P-S28) *(C) Doxorubicin treatment leads to cyclin B1 accumulation.* Mock treated and 200 nM doxorubicin treated cells were harvested at 24 hours post treatment and blotted using antibodies directed against cyclin B1 and actin. *(D) MVM infection results in reduction of cyclin B1 RNA at 24 hpi.* Northern blot analyses comparing RNA from parasynchronized mock and MVM infected cells at 24 hpi. Ethidium bromide stained 18s rRNA served as a loading control. *(E) Time course analyses comparing RNA and protein from mock and infected cells late in infection.* Samples harvested at the indicated time points were processed for western blotting (panels a to e, using antibodies described in Fig. 4A), and RNAse protection assays (RPA, panels f and g) using probes against murine actin and cyclin B1. * - nocodazole added at 19 hpr.

These results suggested that the kinase component of the cyclin B1-CDK1 complex was likely not the critical regulatory target for sustaining the MVM-induced G2 cell cycle block at later time points during infection, and so we turned our attention to cyclin B1 itself. Following MVM infection of para-synchronized murine A9 cells, cyclin B1 levels were seen to accumulate at 18 h pi, increasing through the 24 h time point (Figure 5, panel e, lanes 4 and 5). However, remarkably, by 33 h pi, there was a dramatic reduction in the accumulated levels of cyclin B1 (Figure 5A, panel e, compare lanes 5 and 6). This was unexpected as cyclin B1 expression normally peaks in G2 in cycling cells [23]. Because cyclin B1 is a required cofactor for the G2 to M transition, its depletion would account for the sustained loss of kinase activity we observed (Figure 4, panels a and i, lanes 1 and 2), and the apparent failure of dephosphorylated CDK1 (see Figure 5A, panel b, lane 6) to promote mitotic entry in MVM infected cells. In these experiments total CDK1 expression became detectable around 12 h post release (when cells had begun to cycle into S-phase) (Figure 5A, panel c, lane 3), while the inhibitory tyrosine-15 phosphorylation was again lost by 33 h pi (Figure 5A, panel b, lane 6). NS1 expression was used as a marker for infection (Figure 5A, panel a), and cyclin A levels indicated entry into S-phase and was sustained thereafter (Figure 5A, panel d, lanes 3–6).

To confirm that MVM infected cells in which cyclin B1 was lost had not proceeded into mitosis, we performed a nocodazole trap experiment. Cells that are blocked prior to mitosis can be differentiated from normal cycling cells by immunostaining with an antibody to histone H3 phosphorylated on serine 28, which is a marker for cells present in mitosis [42]. As expected, uninfected cells (which only had a relatively low percentage of cells in mitosis) showed little histone H3 phosphorylation (Figure 5B, lane 1), while nocodazole treatment resulted in an increase in phosphorylated histone H3 (Figure 5B, lane 3), consistent with an accumulation of cells in mitosis. In contrast, MVM infected cells did not exhibit histone H3 phosphorylation either in the absence (Figure 5B, lane 2), or presence (Figure 5B, lane 4), of nocodazole, indicating that MVM infected cells were blocked prior to mitotic entry, presumably due to the absence of cyclin B1. Typically, the highest amounts of cyclin B1 are found in G2/M cells since cyclin B1 transcription begins in S-phase and peaks at the G2/M border [23]. In contrast to MVM infected cells, arrest of uninfected A9 cells at the G2/M border by doxorubicin resulted in dramatic elevation of cyclin B1 protein compared to mock asynchronous cells (Figure 5C, compare lanes 1 & 2). This result confirmed that the loss of cyclin B1 observed following MVM infection was a specific, virally-induced event, and likely the cause rather than the consequence of the cell cycle block.

A reduction in cyclin B1 mRNA was also apparent during MVM infection as early as 24 hpi (Figure 5D, compare lanes 1 & 2). To compare cell cycle alterations with mock infected cells in parallel also at later time points, mock cells were treated with nocodazole at 19 h post release to prevent entry into the next cell cycle phase. Cells were processed for western blotting and RNAse protection assays in parallel. The results demonstrated a correlation between the reduction in cyclin B1 protein and RNA both at 25 h (Figure 5E, lanes 5 & 6, compare panels e and f) and 32 h (Figure 5D, lanes 7 & 8, compare panels e and f) after release.

#### MVM infection induced premature nuclear entry and recruitment to APAR bodies of cyclin B1

**Figure 6:**
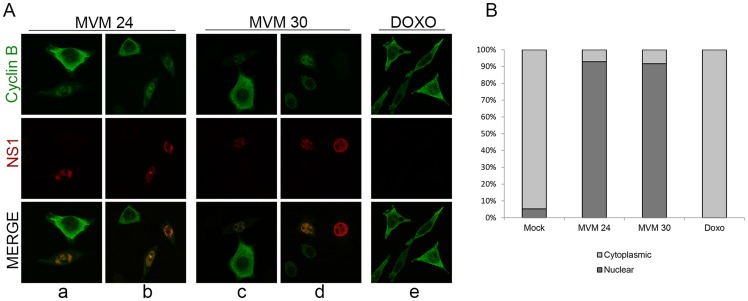
MVM infection induces premature nuclear entry, recruitment into APAR bodies and eventual loss of cyclin B1. *(A) Mislocalization of cyclin B1.* Panels a to d; para-synchronized A9 cells were infected with MVMp (MOI of 10) for 24 or 30 hours before being fixed and processed for immunofluorescence. APAR bodies were detected with antibodies to NS1. Representative images are shown. G2 cells (identified by prominent cytoplasmic staining of cyclin B1) are shown juxtaposed to infected cells. In infected cells which showed cyclin B1 staining, cyclin B1 was nuclear and observed within distinct foci which co-localized with APAR bodies. Also, cyclin B1 staining intensity was reduced in many infected cells (compared to G2 cells, see figure 6C) and was completely absent in some (see Figure 6D, compare infected cell on the far right with the one adjacent). All images were captured using an objective of 63×. Panel e; A9 cells were treated with 200 nM doxorubicin (doxo) for 28 hours and processed as above. *(B) Quantitation of the intracellular distribution of cyclin B1.* Mock, doxorubicin-treated and MVM infected A9 cells from the experiment in (A) and an additional experiment were quantified to indicate the cellular region showing predominant cyclin B1 staining. Approximately 100 cells with detectable cyclin B1, in 7 randomly selected fields, were scored in an unbiased manner and plotted. Mock cells in G0/G1 as well as MVM-infected cells with significantly reduced cyclin B1 staining were not included in the scoring.

Under normal conditions, both the expressed levels and localization of cyclin B1 are tightly regulated. In normal cycling cells, cyclin B1 levels progressively increase in the cytoplasm of S and G2 phase cells and only begin to accumulate in the nucleus just prior to nuclear envelope breakdown. It has recently been shown that nuclear import of the cyclin B1/CDK1 complex is dependent on its own kinase activity [43], [44]. At early times during MVM infection, prior to the loss of cyclin B1, we have shown that the complex was inactive due to the inhibitory phosphorylation of CDK1 (Figure 4, panels a and i, lanes 1 and 2), thus, we expected that at these times cyclin B1 would remain cytoplasmic. However, surprisingly, at 24 h post MVM infection, when 70–80% of cells were infected, we observed that the cyclin B1 that was present displayed a nuclear localization in approximately 90% of infected cells (see Figure 6B), and in many cells showed co-localization with MVM NS1 in APAR replication centers (Figure 6A, panels a and b). At this time point we also found many infected cells that showed reduced cyclin B1 staining (see Figure 6A, panel a). Consistent with the results shown in Figure 5A, by 30 h pi there was very little staining of cyclin B1 apparent, with some cells exhibiting undetectable cyclin B1 in these assays (Figure 6A, panel d). In contrast, doxorubicin treated cells blocked in G2 exhibited both an increased accumulation and cytoplasmic localization of cyclin B1 (Figure 6A, panel e) which was restricted to the cytoplasm in all cells with detectable cyclin B1 expression (Figure 6B). Together, these results suggest that MVM infection led to an early mis-localization of cyclin B1. It is not known whether mis-localization of cyclin B1 at early times during infection is a necessary prelude to its eventual loss.

## Discussion

DNA viruses induce cellular DDRs that can present a block to infection that must be overcome, or alternatively can be utilized to viral advantage [45]. Many viruses, including parvoviruses, have been shown to induce DDR-dependent cell cycle alterations in infected cells, employing varying mechanisms [46], [47]. Due to their small genome sizes, parvoviruses do not encode their own polymerases, and must rely on cellular proteins in order to replicate their genomes [3]. A few hours after S phase entry, viral replication commences and soon thereafter, a DNA damage response characterized by phosphorylation of a number of cellular DDR-related proteins is initiated [7]–[9]. Viral replication is required for full induction of the MVM-dependent DDR; however, the specific trigger of this response is not yet clear. Coincident with viral replication and an ongoing DDR, cellular replication begins to decline. Infected cells undergo a transient S-phase block and eventually become fully arrested prior to mitosis [1], [7], [21]. A poorly understood feature of autonomous parvovirus replication is that it is sustained for long periods of time in a pre-mitotic nuclear environment following cessation of cellular DNA replication [12].

MVM genome replication is a source of ongoing DDR induction yet p21 and Chk1, major players typically associated with S-phase and G2-phase cell cycle arrest in response to diverse DNA damage stimuli, are either down-regulated, or inactivated, respectively, during infection [7], [33]. We have shown that MVM infection induced a cell cycle block independently of these two proteins via a two-step mechanism which is unlike that seen following other DNA-damaging agents or virus infection. The Chk2 protein was first activated and recruited to MVM replication centers during infection. Chk2 activation was necessary to induce a transient S-phase block associated with CDC25A degradation which was necessary for full levels of viral replication. Chk2 activation and CDC25A loss, however, were not sufficient to induce the dramatic G2 arrest seen following MVM infection. While the Y15 phosphorylation of CDK1 that normally inhibits entry into mitosis was lost as infection progressed, the MVM induced DDR resulted in the near-complete depletion of cyclin B1, thus directly inhibiting cyclin B1-CDK1 complex function. This was likely to be the main cause of the more permanent G2 arrest that ensured that infected cells did not proceed into mitosis.

The intra-S-phase reduction in cellular DNA replication is a general response to DNA damage stimuli that helps affected cells to correct replication defects before proceeding to G2 and M [35]. Our data suggests that parvoviruses may exploit this response in order to prime the cell to support its replication. Upon DDR induction in MVM infected cells, Chk2 was phosphorylated by ATM. There was a pan-nuclear increase in phospho-Chk2 staining and, importantly, accumulation of the phosphorylated protein in APAR bodies where viral replication takes place. Chk2 activation resulted in proteasome-mediated CDC25A degradation. Since CDC25A phosphatase is necessary for activating CDK2 [40], loss of CDC25A in MVM infected cells likely led to reduced CDK2 activity, which would be predicted to inhibit cellular DNA replication machinery [48] and cell cycle progression [49]. Additionally, Chk2 activity was recently shown to directly inhibit the replicative helicase complex, providing an alternative basis for Chk2-mediated S-phase arrest [50]. Our inhibition of Chk2 kinase activity thus abrogated the virus-mediated S-phase arrest resulting in a subsequent reduction in viral replication.

Complete inhibition of CDK2 during S-phase would likely prevent viral replication. But, in order to have complete inhibition of CDK2 activity and a total S-phase block, both the ATM/Chk1-Chk2/CDC25A pathway and the ATM/Nbs1/SMC1 pathway must be inactivated [35], [36], [51], [52]. We did not observe significant activation of SMC1 during MVM infection. Indeed in preliminary experiments we have found reduced, but not complete, inactivation of CDK2 activity following infection (Adeyemi and Pintel, unpublished). Following IR treatment, inactivation of either the Chk2-CDC25A pathway or the SMC1 pathway alone results in a partial radio-resistant DNA synthesis (RDS) phenotype - a scenario in which a reduced level of DNA synthesis still takes place following radiation treatment [51]. We recently found that the potent CDK inhibitor p21, which inhibits repair synthesis, is targeted for degradation during MVM infection [33], and our earlier work has shown that complete abrogation of CDK2 activity via roscovitine treatment reduced virus replication [33]. Thus, it is possible that activation of Chk2 but not SMC1 during MVM infection may have allowed the low levels of CDK2 activity necessary to maintain synthesis of viral DNA, while still limiting cellular DNA replication in these cells.

In addition to transiently blocking cells in S phase, parvovirus infection results in a marked pre-mitotic cell cycle arrest [7], [21]. Activity of the cyclin B1/CDK1 complex, which is required for mitotic entry, is dependent on the levels of cyclin B1, its localization in the cell, and regulatory phosphorylation of CDK1 [23]. Typically, maintenance of the inhibitory phosphorylation of CDK1 serves to halt cells in G2 phase following DNA damage stimuli [30]. The inactive complex is retained in the cytoplasm until the source of the damage stimulus is removed [23]. As expected, at late times during MVM infection kinase assays demonstrated that CDK1 activity was inhibited; surprisingly however, we found that the inhibitory phosphorylation of CDK1 was lost. Instead, we observed a dramatic reduction in cyclin B1 levels at this time, ensuring inactivity of the cyclin B1/CDK1 complex and a block to mitotic entry. Arrest of uninfected A9 cells at the G2/M border by doxorubicin resulted in dramatic elevation of cyclin B1 protein suggesting that the loss of cyclin B1 observed following MVM infection was a specific, virally-induced event, and likely the cause rather than the consequence of the cell cycle block.

Failure to sustain the inhibitory phosphorylation of CDK1 may have been due to absence of Chk1 activation. Chk1 exerts its inhibitory effects on CDK1 *via* inhibitory phosphorylation of CDC25C [29], [40], however we have been unable to detect Chk1 activation in MVM infected cells. Chk2 has also been shown to phosphorylate CDC25C *in vitro* [31]; however, our data suggest that Chk2 activity is unable to sustain a permanent G2 arrest during MVM infection. Ectopic expression of the MVM NS1 protein by itself led to an increase rather than decrease in cyclin B1 levels (data not shown), suggesting that an event mediated by viral replication and induction of the DDR triggered the reduction of cyclin B1 levels in MVM infected cells. Reduction in cyclin B1 appeared to correlate with a prior depletion of cyclin B1 mRNA. The cause of cyclin B1 loss is not yet clear. In preliminary experiments, siRNA depletion of p53 partially restored cyclin B1 levels, suggesting that p53 activation may be involved in cyclin B1 RNA depletion (Adeyemi, Fuller, and Pintel, in preparation).

Typically, cyclin B1 levels progressively accumulate in cells during S-phase, peaking in late G2 and early mitosis [23]. MVM infection did not appear to prevent the initial increase in cyclin B1 protein levels; cyclin B1 was detected early and increased through 24 h pi before beginning to decrease. Depletion of cyclin B1 mRNA, however, occurred as early as 24 h after infection. Interestingly, in cells that had not yet lost cyclin B1, we observed nuclear localization of cyclin B1 and co-localization with NS1 in APAR replication bodies. This was unexpected because cytoplasmic retention of cyclin B1 is a general mechanism governing cell cycle arrest during infection by various viruses and DNA damage stimuli [46], [47]. Furthermore, while it has recently been shown that that cyclin B1/CDK1 activation is necessary for nuclear localization of the complex [43], [44], [53], in MVM infected cells cyclin B1 showed nuclear localization even though the kinase remained inactive. It is not yet clear whether the mis-localization of cyclin B1 at early times during infection is a necessary step in its subsequent depletion.

There is substantial evidence indicating that the sustained S/G2 cell cycle block seen following MVM infection is essential for its replication. Abrogation of MVM-induced DNA damage signaling by caffeine and ATM inhibitors, which by themselves did not affect cell cycle distribution of mock infected cells, led to a significant reduction in both viral replication and virus-induced S/G2 arrest [7]. Here we have shown that inhibition of Chk2, whose activity appears to function after the onset of MVM replication (as evidenced by the initial accumulation of NS1), and which blocks subsequent S-phase progression, significantly reduced MVM replication without substantially affecting the proportion of cells blocked in G2 phase. We have not excluded, however, that Chk2 activity affects MVM replication in ways independent of an S-phase block. Similarly, a recent report has also shown that S-phase arrest following MVC infection is critical for its replication [18]. We assume that depletion of cyclin B1 is also a necessary feature of MVM infection; however, how reduced cyclin B1 levels facilitate infection is not yet clear. A number of other DNA viruses have recently been shown to exploit a G2 arrest to promote their replication [16], [46], [54].

In conclusion, we have shown that during MVM infection Chk2 activation led to a transient S-phase block which was associated with CDC25A degradation and was necessary for viral replication. Chk2 activation and CDC25A loss were alone not sufficient to sustain the G2 arrest seen following MVM infection. Rather, although the phosphorylation of CDK1 that normally inhibits entry into mitosis was lost as infection progressed, the MVM-induced DDR resulted first in a targeted mis-localization and then significant depletion of cyclin B1, thus directly inhibiting cyclin B1-CDK1 complex function and preventing mitotic entry. MVM infection thus uses a novel strategy to ensure a pseudo S-phase, pre-mitotic, nuclear environment for sustained viral replication.

## Materials and Methods

### Cell lines, viruses and virus infections

Murine A9 and human NB324K cells were propagated as previously described. Wild-type MVMp was propagated following transfection of the viral infectious clone in NB324K cells and titered by plaque assay on A9 cells as previously described [7]. Infections were carried out at an MOI of 10 unless otherwise indicated. Where indicated, reinfection was blocked by addition of viral capsid neutralizing antibodies to the media.

### Cell synchronization and drug treatments

A9 cells were para-synchronized in G0 by isoleucine deprivation. Unless otherwise indicated Doxorubicin (Sigma) was used at a final concentration of 200 nM. Chk2 inhibitor II was obtained from Sigma and used at a final concentration of 10 µM. Nocodazole was obtained from Calbiochem and used at a final concentration of 150 ng/ml, and controls were treated with the DMSO vehicle.

### siRNA transfections

ON-TARGET plus SMART pool siRNAs directed against mouse Chk2 (cat # L-0406034-00) was obtained from Dharmacon. A9 cells plated in isoleucine-deprived media in 60 mm dishes were transfected at the day of plating with 40 nm of siRNA using HiPerfect transfection reagent (Invitrogen). Transfections were repeated 24 hours later and the next day the cells were released into complete media and processed as described in the figure legends.

### Antibodies

Commercially available antibodies obtained from Cell Signaling against the indicated proteins were: Cdc2/CDK1 (Cat # 9112S), CDK1-P-Y15 (Cat # 4539S), Histone H3-P-S28 (Cat # 9713S), p53-P-S15 (Cat # 9284), SMC1-P-957 (Cat # 4805S), SMC1 (Cat # 4802S), CDC25A (Cat # 3652S) and Chk2-P-T68 (Cat # 2661S). Antibodies obtained from Santa Cruz Biotechnology were CDC25A F-6 (Cat # sc -7389), CDC25C H-150 (Cat # sc-5620), Cdc2 (Cat # sc-54). Others were against actin (Cat. 109 # MA515739, Pierce), RPA32-P-S4/8 (Cat # A300-245A, Bethyl), cyclin A (Cat # 06-138, Upstate) γH2AX (Cat # 05-636, Millipore), Chk2 Clone 7 (Cat # 05-649, Millipore), cyclin B1 (Cat. # 05-373, Millipore), and CDK1 (Cat. #Ab18, Abcam). Secondary antibodies have previously been described. Additional antibodies used for immunofluorescence include: cyclin B1 (Cat # 4138S, Cell signaling), Alexa 488 (anti-mouse Cat # A11029, anti-rabbit Cat # A11034, Invitrogen) and Alexa 568 (anti-mouse Cat # A11031, anti-rabbit Cat # A11036, Invitrogen). NS1 CE10 and NS1 91W were described previously [7].

### Immunoblot analyses

Cells grown and infected in 60 mm dishes were harvested and lysed in modified RIPA buffer containing 20 mM Tris HCL pH 7.5, 150 mM NaCL, 10% glycerol, 1% NP-40, 1% sodium deoxycholate, 0.1% SDS, 1 mM EDTA, 10 mM trisodium pyrophosphate, 20 mM sodium fluoride, 2 mM sodium orthovanadate and 1× protease inhibitor cocktail (Sigma). Alternatively, cells were lysed in 2% SDS lysis buffer directly on cell culture dishes as previously described. Protein concentrations were quantified by Bradford assay and equal amounts of lysates were loaded in wells and used for western blot analyses as previously described [7].

### Kinase assays

Kinase assays were performed using a previously described protocol [55]. Briefly, CDK1 was immunoprecipitated from equal amounts of infected or drug-treated A9 cell lysates using 4 µg of CDK1 antibody (Cat # Ab18, Abcam). Histone H1 (Millipore) was used as a substrate for kinase assays.

### Immunofluorescence

For immunofluorescence, NB324K cells or para-synchronized A9 cells were grown on glass coverslips in 35 mm dishes and infected with MVMp using an MOI of 10. After 24–30 hr, cells were washed with PBS, fixed with 4% paraformaldehyde for 15 min and extracted with 0.5% Triton X-100 in PBS for 10 min. After blocking in PBS containing 3% BSA, cells were stained with the indicated antibodies. Nuclei were visualized by staining with either DAPI or TOPRO3. The coverslips were mounted in Fluoromount-G (Southern Biotech) and images were acquired using a Zeiss LSM 510 Meta confocal microscope. All images were captured using an objective of 63×.

### Cell cycle analyses

An hour before infection, cells were pre-treated with the 10 µM Chk2 inhibitor II or vehicle (DMSO) control. Cells were then mock infected or infected at an MOI of 10. After 24 hours, cells were harvested and fixed in 4% formaldehyde for 15 min at room temperature. Alternatively, cells were fixed in 70% ethanol for 15 min on ice. Cells were then pelleted, washed in PBS and resuspended in 50 µg/ml propidium iodide solution containing 0.1 mg/ml RNAase A as well as 0.05% Trition X-100 for 40 min at 37°C. Cells were resuspended in PBS and flow cytometry was performed using FACScan (BD Biosciences). Data were analyzed using Summit software (Beckman Coulter).

### Analysis of viral DNA

Cell pellets from 60 mm dishes were split in two, with one half used for western blot analysis and the other half for Southern blot analysis. Southern blots were carried out as previously described using whole MVM genome probes. Loading of DNA samples was normalized using a nanodrop spectrophotometer, and results and quantifications were standardized using probes against mitochondrial DNA as described [56].

### RNAse protection assay (RPA)

RPA's were performed as previously described [57]. Total RNA was isolated using Trizol reagent (Invitrogen). Murine cyclin B1 cDNA was obtained from Origene. Nucleotide 1 to 180 of murine cyclin B1 cDNA was cloned into pGEM3Z to make an antisense probe.

### Northern blots

Total RNA was extracted using Trizol reagent. Northern blots were performed as previously described [58]. Cyclin B1 was detected by probing with ^32^P-labeled full length murine cyclin B1 cDNA.
